# Ginseng increases Klotho expression by FoxO3-mediated manganese superoxide dismutase in a mouse model of tacrolimus-induced renal injury

**DOI:** 10.18632/aging.102137

**Published:** 2019-08-10

**Authors:** Sun Woo Lim, Yoo Jin Shin, Kang Luo, Yi Quan, Sheng Cui, Eun Jeong Ko, Byung Ha Chung, Chul Woo Yang

**Affiliations:** 1 Convergent Research Consortium for Immunologic Disease, Seoul St. Mary’s Hospital, College of Medicine, The Catholic University of Korea, Seoul, Republic of Korea; 2 Transplant Research Center, College of Medicine, The Catholic University of Korea, Seoul, Republic of Korea; 3 Division of Nephrology, Department of Internal Medicine, Seoul St. Mary’s Hospital, College of Medicine, The Catholic University of Korea, Seoul, Korea

**Keywords:** ginseng, Klotho, oxidative stress, tacrolimus, MnSOD, mitochondria

## Abstract

The antioxidant function of Klotho is well-documented as a regulatory factor implicated in countering the aging process. This study investigated whether ginseng upregulates Klotho and its antiaging signaling in a setting of calcineurin inhibitor-induced oxidative stress. Although tacrolimus treatment reduced Klotho level in the serum and kidney, ginseng treatment was found to reverse the levels. Tacrolimus-induced oxidative stress was reduced by ginseng treatment, with functional and histological improvements. Effect of ginseng on Klotho-induced manganese superoxide dismutase signaling pathway during tacrolimus treatment in mice revealed that ginseng suppressed phosphatidylinositol 3-kinase/serine-threonine kinase Akt-mediated phosphorylation of forkhead box protein O3a and promoted the binding of forkhead box protein O3a to manganese superoxide dismutase promoter. In the mitochondria, ginseng reduced mitochondrial reactive oxygen species production, mitochondrial membrane potential, and oxygen consumption rate, whereas blocking phosphatidylinositol 3-kinase activity with LY294002 enhanced them. These findings together suggested that ginseng attenuated tacrolimus-induced oxidative stress via signaling between Klotho and the phosphatidylinositol 3-kinase/serine-threonine kinase Akt/forkhead box protein O3a-related antioxidant pathway.

## INTRODUCTION

Klotho is a recently identified antiaging gene [1]. It encodes a single-pass trans-membrane protein presented primarily in the kidney [2]. Although its reduced expression induces an aging-like syndrome in mice, its overexpression extends the life span of an animal [2–4]. Klotho protein is predominantly expressed in the distal tubule of the kidney and in the choroid plexus in the brain [4–6]. Several researchers reported the antioxidant function of Klotho [7–11]. Klotho increases the activity of forkhead box protein O transcription factor 3a (FoxO3a), which functions as a negative regulator of mitochondrial reactive oxygen species (ROS) production by inhibiting phosphatidylinositol 3-kinase (PI3K) and serine-threonine kinase Akt (AKT) [12], thereby suggesting that Klotho might suppress ROS-related oxidative stress. Hence, the antiaging function of Klotho may involve ROS and its downstream signaling pathway.

The use of calcineurin inhibitors (CNI) has led to major advance in the field of transplantation, with excellent short-term outcomes. However, chronic nephrotoxicity is a major cause of chronic allograft dysfunction and allograft failure in transplant recipients [13–15]. Experimental studies showed that long-term administration of CNI causes progressive renal failure [16–20], which is similar to that in aging kidney. Recent studies revealed that oxidative stress caused by ROS plays an important role in CNI-induced nephrotoxicity [9, 11, 20]. Thus, we hypothesized that CNI-induced oxidative stress may accelerate the aging process in the kidney and evaluated the role of Klotho in CNI-induced oxidative stress. Using an experimental model of chronic CNI nephropathy, we found that CNI treatment decreased Klotho protein in a dose- and time-dependent manner, and Klotho deficiency aggravated CNI-induced oxidative stress. Administration of recombinant Klotho protein decreased CNI-induced oxidative stress by enhancing manganese superoxide dismutase expression via the phosphatidylinositol 3-kinase-Akt-Forkhead box protein O pathway. These findings suggested that Klotho had a profound effect on FoxO activity and oxidative stress caused by CNI [10].

Ginseng, a well-known traditional medicine and tonic, has been used as panacea or to promote longevity [21]. Abundant evidence suggests that oxidative stress plays a central role in the process of biological aging [22]. Excessive oxidative stress leads to cell death and mitochondrial dysfunction [23]. Some research indicated that ginseng extracts improve learning and memory in normal, aged, or brain-damaged animals [24, 25]. In 1991, Bernhard et al., reported that ginseng enhances the age-dependency of learning ability in female rats, as determined by the passive avoidance test [26]. Nevertheless, there is mounting evidence of the antiaging effect of ginseng, but little research on Klotho is published. Therefore, in this study, we investigated whether the FoxO3a-mediated antioxidant property of Klotho through inhibition of the PI3K/AKT pathway is closely associated with the antiaging activity of ginseng.

## RESULTS

### KRGP ameliorates Tac-induced renal dysfunction and fibrosis

**Figure 1:**
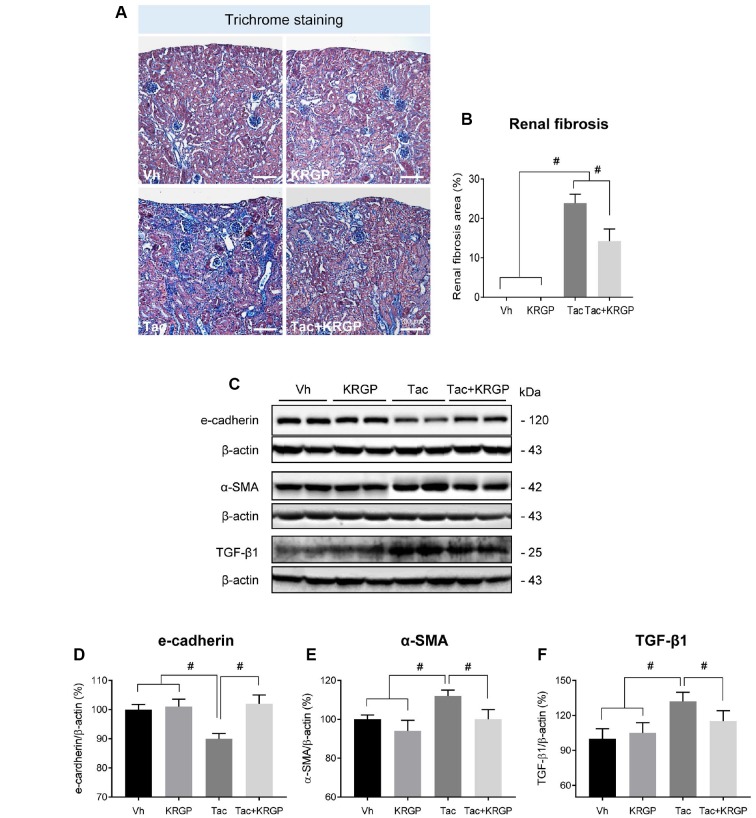
**Effect of KRGP administration on Tac-induced renal fibrosis in a mouse model.** (**A** and **B**) Histological analysis in the renal cortex in mice treated with Tac for 4 weeks showed striped tubulointerstitial fibrosis, mononuclear cell infiltration, and tubular atrophy. KRGP treatment significantly reduced these damages compared with those caused by Tac treatment. (**C**–**F**) Immunoblotting analysis of e-cadherin, α-smooth muscle actin (α-SMA), and transforming growth factor β1 (TGF-β1) in the renal cortex. The relative optical densities of bands in each lane were normalized to that of each β-actin band in the same gel. Note that the expression of e-cadherin was restored and that of α-SMA and TGF-β1 was decreased after combined treatment with KRGP. Bar = 100 μm. Data are presented as mean ± SE. *n* = 8. ^#^P < 0.05.

Table 1 shows alterations in functional parameters after four weeks of Tac and Korean Red Ginseng Powder (KRGP) treatment. Tac treatment reduced body weight and serum Klotho levels, and increased serum creatinine (Scr) levels compared to that in the Vh group; however, co-treatment with KRGP significantly restored both Scr and Klotho levels compared to that in the Tac group. KRGP significantly reduced the extensive Tac-induced interstitial fibrosis, as shown in Figure 1A and 1B. On molecular basis, Tac treatment reduced the expression levels of e-cadherin and increased those of α-smooth muscle actin (α-SMA) and transforming growth factor-beta 1 (TGF-β1); the levels were restored by treatment with KRGP (Figure 1C–1F).

**Table 1:** Effect of administration of KRGP on Tac-induced renal injury in mice.

	**Vh**	**KRGP**	**Tac**	**Tac+KRGP**
Body weight (g)	26.1 ± 1.0	25.7 ± 0.9	24.3 ± 1.1^1^	25.0 ± 0.8^3^
Scr (mg/dL)	0.31 ± 0.02	0.28 ± 0.02^1^	0.50 ± 0.02^1,2^	0.43 ± 0.02^3^
Plasma Klotho (pg/mL)	1.96 ± 0.36	2.05 ± 0.50	0.71 ± 0.03^1,2^	1.06 ± 0.17^3^

Vh, vehicle; KRGP, Korean Red Ginseng Powder; Tac, tacrolimus; Scr, serum creatinine. The values shown are the mean ± SE (*n* = 8). ^1^P < 0.05 vs. Vh; ^2^P < 0.05 vs. KRGP; ^3^P < 0.05 vs. Tac.

### KRGP reduces oxidative stress and apoptosis in Tac-treated mice

**Figure 2:**
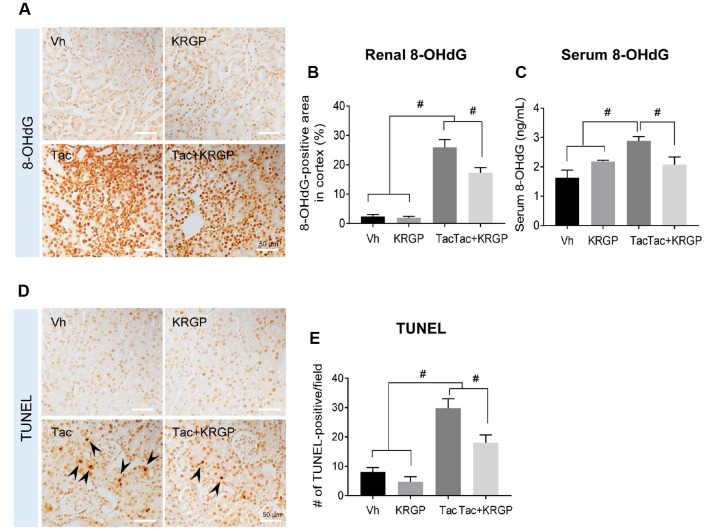
**Effect of KRGP administration on Tac-induced the expression of 8-OHdG and TUNEL in a mouse model.** (**A** and **B**) Representative images and immunohistochemical assay results for 8-OHdG in tissue sections from mouse kidney. (**C**) 8-OHdG level in mouse serum. (**D**–**E**) Representative images and quantification of TUNEL assay in tissue sections from mouse kidney. Bar = 50 μm. Data are presented as mean ± SE. *n* = 8. ^#^P < 0.05.

Figure 2 presents renal 8-hydroxy-2′deoxyguanosine (8-OHdG; a marker of oxidative DNA) immunoreactivity and serum 8-OHdG levels (Figure 2A–2C). Kidney tissue and serum 8-OHdG level were dramatically augmented by Tac treatment, but were then restored by the addition of KRGP. We also explored whether KRGP protects against Tac-induced apoptosis, which is a common cell death pathway following Tac treatment. Higher number of terminal deoxynucleotidyl transferase dUTP nick end labeling (TUNEL)-positive cells was observed in the Tac group than in the vehicle (Vh) group, which was attenuated by co-treatment with KRGP (Figure 2D and 2E).

### Effect of KRGP on Tac-induced mitochondrial ultrastructure

**Figure 3:**
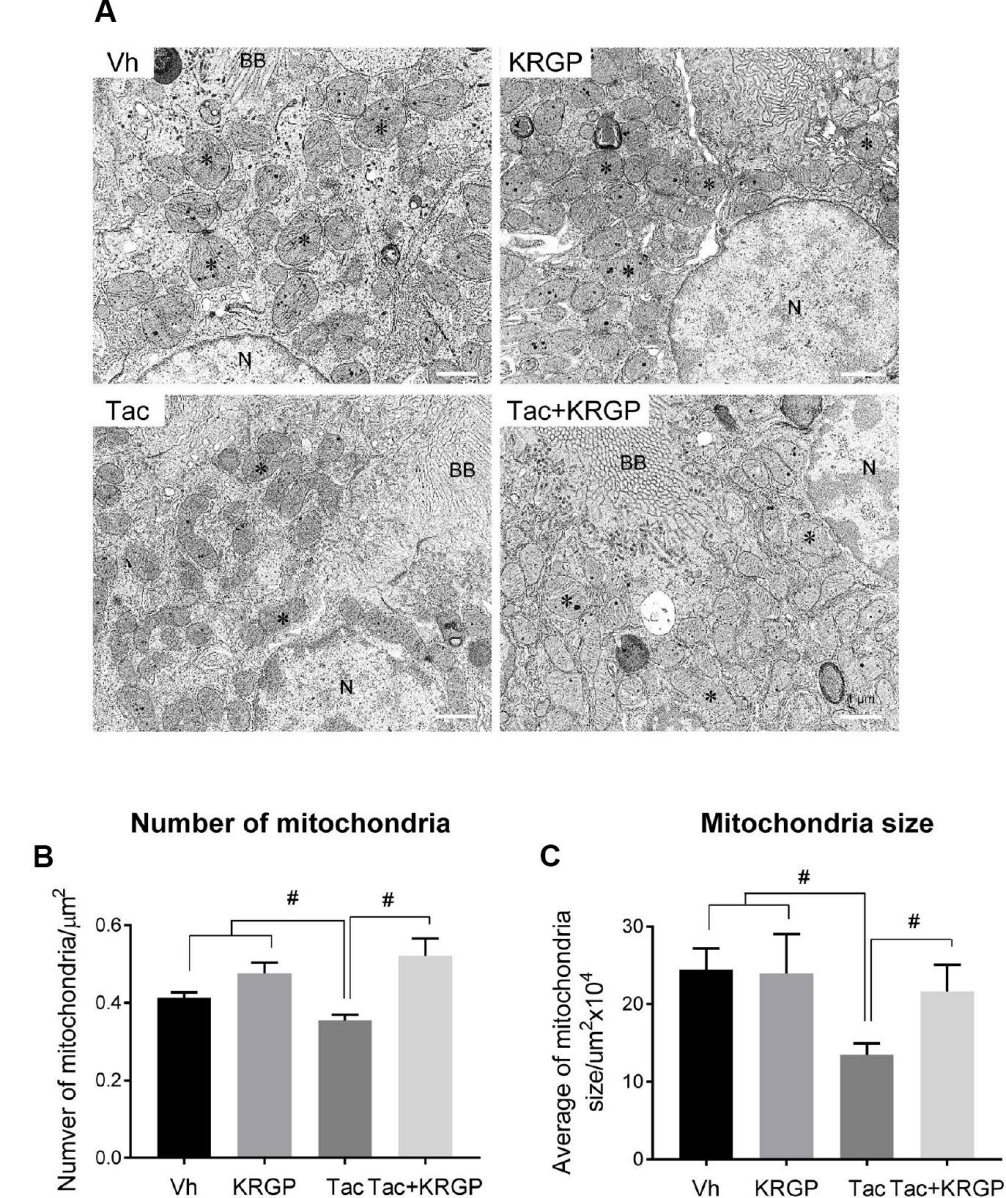
**Effect of KRGP administration on Tac-induced mitochondrial ultrastructure in a mouse model.** (**A**) Representative transmission electron micrographs of mitochondrial ultrastructure in the proximal tubules. Asterisks indicate mitochondria. (**B** and **C**) Quantitative analysis of number and size of mitochondria. BB, brush border. N, Nucleus. Bar = 1 μm. Data are presented as mean ± SE. *n* = 8. ^#^P < 0.05.

Figure 3 shows whether KRGP is closely associated with oxidative stress and apoptosis by regulating mitochondrial function. We observed morphological changes in the mitochondria by electron microscopy. Ultrastructural analysis suggested a reduction in the number and size of mitochondria after Tac treatment, which was eventually reversed by the addition of KRGP (Figure 3A–3C).

### Effect of KRGP on Klotho-induced MnSOD signaling pathway after Tac treatment in mice

**Figure 4:**
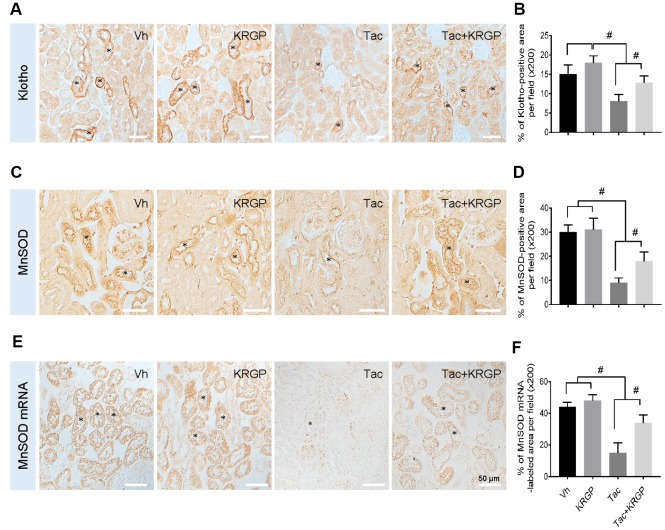
**Effect of KRGP administration on Klotho-induced MnSOD signaling pathway in Tac-induced oxidative stress using immunohistochemical analysis in a mouse model.** Representative images of immunohistochemical analysis and its quantification for Klotho (**A** and **B**) and MnSOD (**C** and **D**), as well as *in situ* hybridization of MnSOD mRNA (**E** and **F**) in the tissue sections from mouse kidneys. Asterisks indicate positive renal tubules for Klotho and MnSOD. Bar = 50 μm. ^#^P < 0.05.

To evaluate whether KRGP induces Klotho and its signaling pathway targeting manganese superoxide dismutase (MnSOD), we performed immunohistochemical assay for Klotho and MnSOD in kidney sections from experimental mice. The images showed that treatment with Tac decreased Klotho expression in the renal tubules, whereas co-treatment with KRGP restored this decrease (Figure 4A and 4D). Moreover, *in-situ* hybridization was performed to detect MnSOD mRNA; although the expression of MnSOD mRNA was remarkably decreased by Tac treatment, the intensity decreased with the addition of KRGP (Figure 4E and 4F).

**Figure 5:**
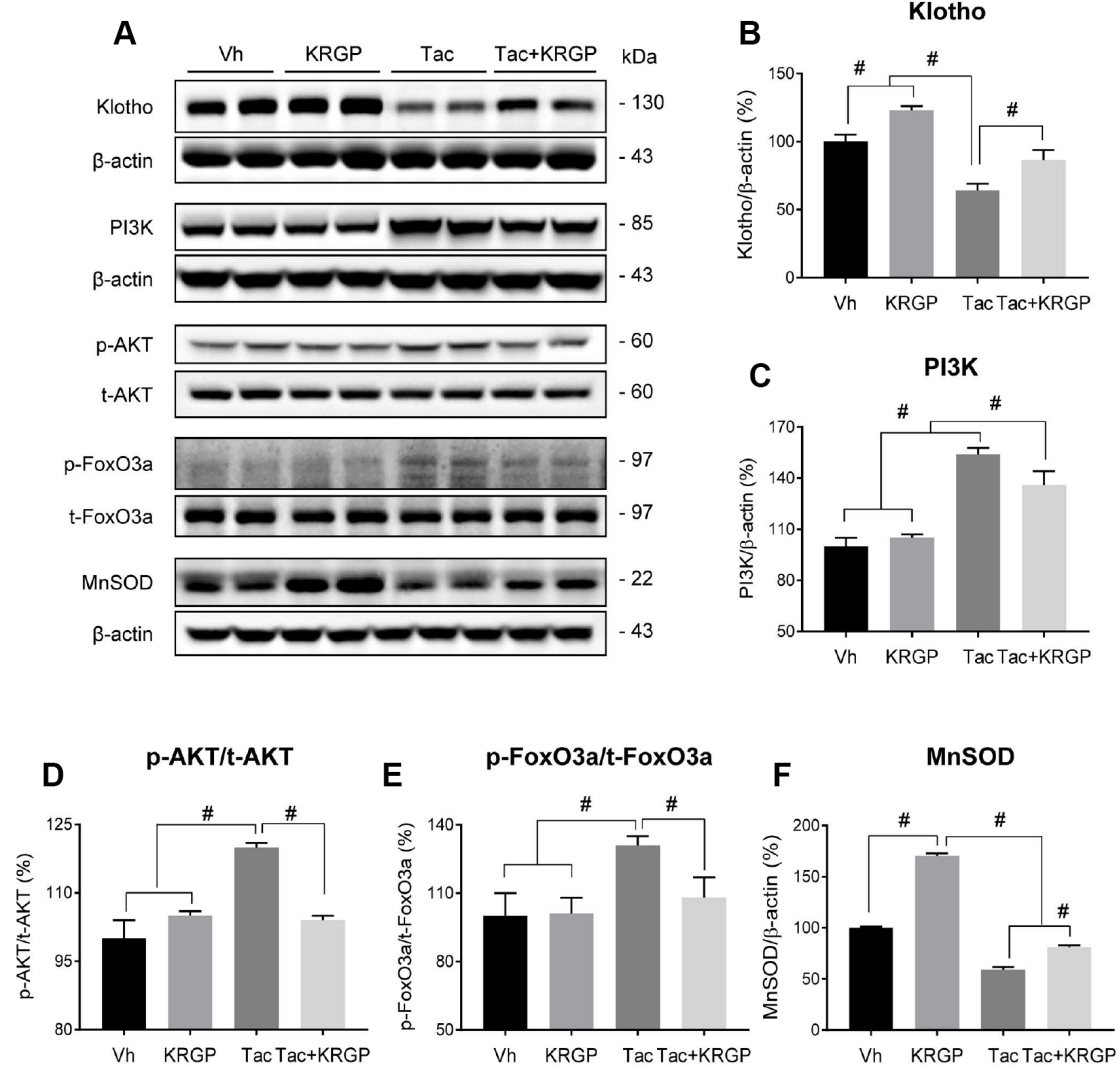
**Effect of KRGP administration on Klotho-induced MnSOD signaling pathway in Tac-induced oxidative stress using immunoblot analysis in a mouse model.** Representative immunoblot image (**A**) and its quantification of Klotho (**B**), PI3K (**C**), p-AKT/t-AKT (**D**), p-FoxO3a/t-FoxO3a (**E**), and MnSOD (**F**) in the renal cortex. The relative optical densities of bands in each lane were normalized to that of each band of β-actin or total form protein in the same gel. Data are presented as mean ± SE. *n* = 8. ^#^P < 0.05.

For further analysis, we examined whether KRGP upregulates the expression of MnSOD through the PI3K/AKT/FoxO3a pathway in renal cortex tissue from mice. Immunoblotting analysis revealed that Tac treatment increased both PI3K and AKT, thereby promoting phosphorylated FoxO3a level (causing deactivation or cytoplasmic retention) and reducing MnSOD transcription level (Figure 5).

### Effect of KRGP on Klotho-induced FoxO3a activation in Tac-treated HK-2 cells

**Figure 6:**
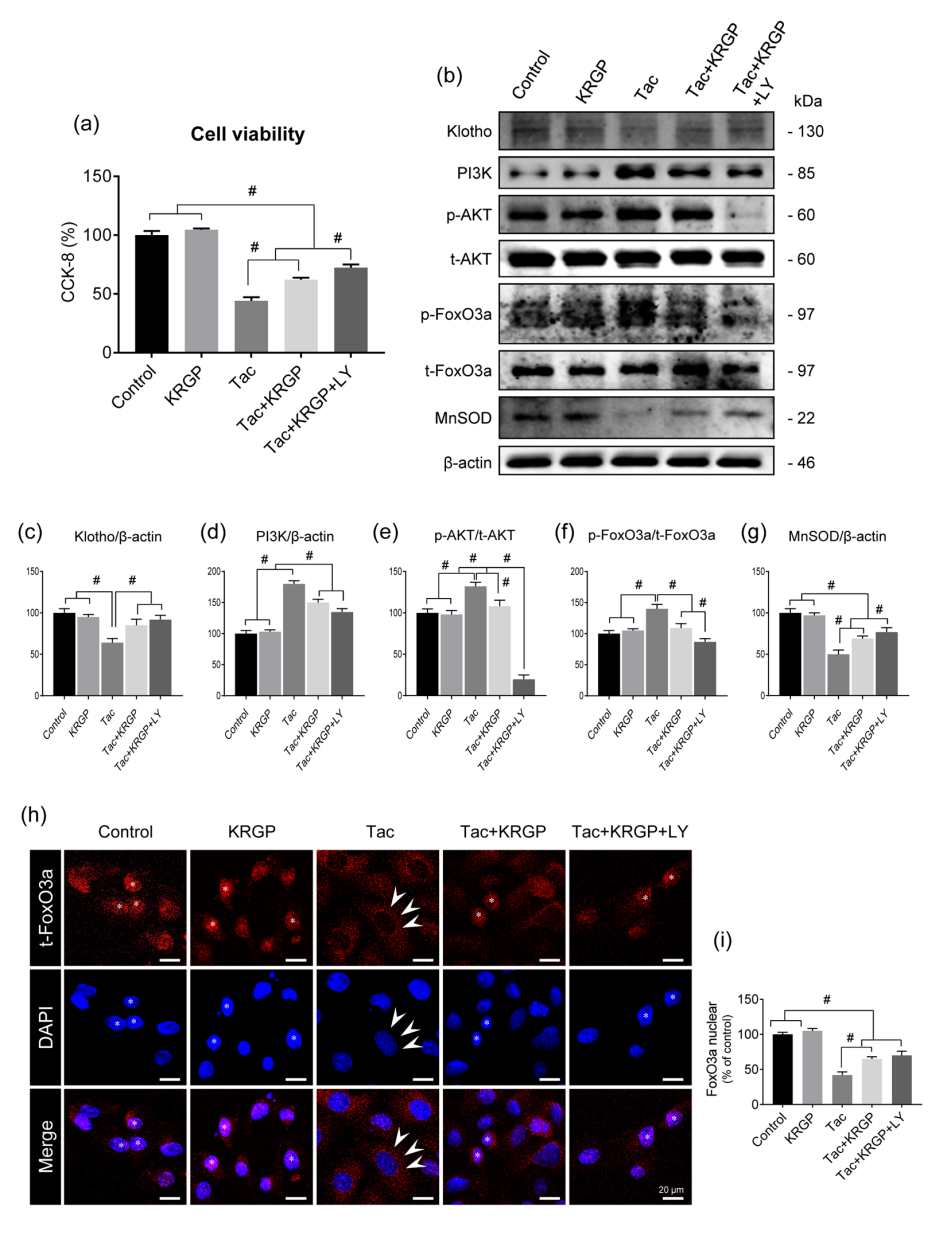
**Effect of KRGP treatment on Klotho-induced MnSOD expression by regulating the PI3K/AKT/FoxO3a pathway in Tac-treated HK-2 cells.** HK-2 cells were seeded in culture plates at 90% confluence. On the next day, the cells were treated with Tac (50 μg/mL) in the absence or presence of 10 μg/mL KRGP and 25 μM LY294002 (LY, PI3K inhibitor) for 12 h. Before the end of the treatment, CCK-8 solution was added to each well for 2 h to measure cell viability. (**A**) Cell-viability assay results of the experimental group. (**B**) Whole-cell lysates were collected after each 12-h drug treatment to measure the protein expression of PI3K, phosphorylated AKT (p-AKT), phosphorylated FoxO3a (p-FoxO3a), and MnSOD. All proteins were normalized to β-actin or total (t) protein controls. (**C**–**G**) Quantitative graph for immunoblot analysis in each group. (**H**) After each 12-h drug treatment, the cells were fixed with fixative and then immunofluorescence was performed with an antibody against t-FoxO3a. Nuclear translocation of t-FoxO3a was observed by confocal microscopy. (**I**) Quantitative graph for nuclear FoxO3a expression in each group. Scale bar = 20 μm. Data are presented as mean ± SE and are representative of at least three independent experiments. ^#^P < 0.05.

Using HK-2 cells, we further examined whether KRGP influences Klotho-induced MnSOD activation via the PI3K/AKT/FoxO3a pathway. We also evaluated the viability of HK-2 cells in the presence and absence of the PI3K-specific inhibitor LY294002 (LY) after treatment with Tac and/or KRGP. As shown in Figure 6A, the protective effect of KRGP increased after LY294002 treatment. Consistent with the *in vivo* results, KRGP reduced FoxO3a phosphorylation by decreasing PI3K/ AKT and increasing MnSOD expression (Figure 6B–6G).

**Figure 7:**
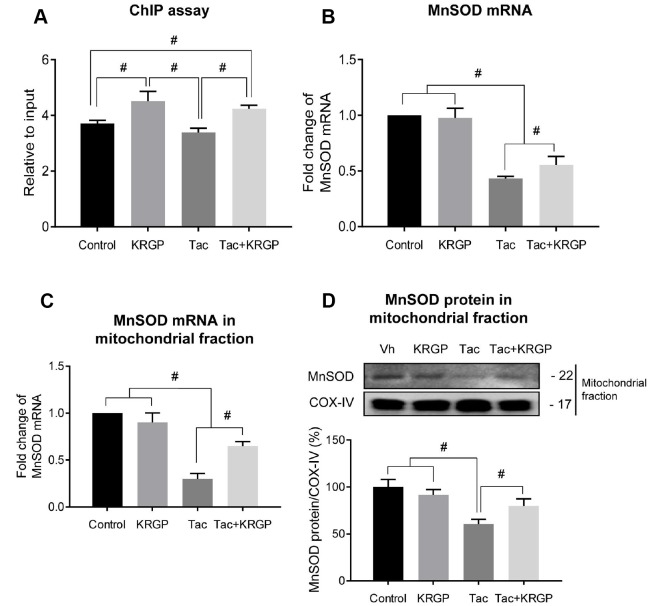
**Effect of KRGP treatment on Klotho-induced FoxO3a binding to the MnSOD promoter in Tac-treated HK-2 cells.** HK-2 cells were seeded in culture plates at 90% confluence. On the next day, the cells were treated with Tac (50 μg/mL) in the absence or presence of 10 μg/mL KRGP. After 12 h, the cells were harvested to detect MnSOD transcription activity after nuclear FoxO3a translocation using a ChIP assay and quantitative real-time PCR (q-PCR) for MnSOD expression. (**A**) The FoxO3a protein/MnSOD promoter complex was analyzed by q-PCR with primer pairs for the MnSOD-promoter region containing a FoxO3a-binding element. (**B**) MnSOD mRNA was detected by q-PCR in whole-cell lysates. (**C** and **D**) MnSOD mRNA and protein expression in mitochondrial fraction from each group. Relative MnSOD protein expression was presented after normalization to CoX-IV expression Relative MnSOD expression was presented after normalization to cyclophilin A expression. Data are presented as mean ± SE and are representative of at least three independent experiments. ^#^P < 0.05.

Next, we performed immunostaining to detect the expression of total FoxO3a (t-FoxO3a), confirming that KRGP induces nuclear translocation of FoxO3a. Cytoplasmic localization of t-FoxO3a after Tac treatment changed to nuclear localization after treatment with KRGP (Figure 6H and 6I). We compared t-FoxO3a immunoreactivity by subjecting the LY-294002-treated groups for positive nuclear staining. Moreover, KRGP treatment increased the binding of FoxO3a to the MnSOD promoter and enhanced MnSOD mRNA level, as observed in the chromatin immunoprecipitation (ChIP) assay (Figure 7A and 7B). Using isolated mitochondrial fraction, we also found that addition of KRGP induced MnSOD expression in the cell mitochondria (Figure 7C and 7D).

### Effect of KRGP on Tac-induced ROS generation in HK-2 cells

**Figure 8:**
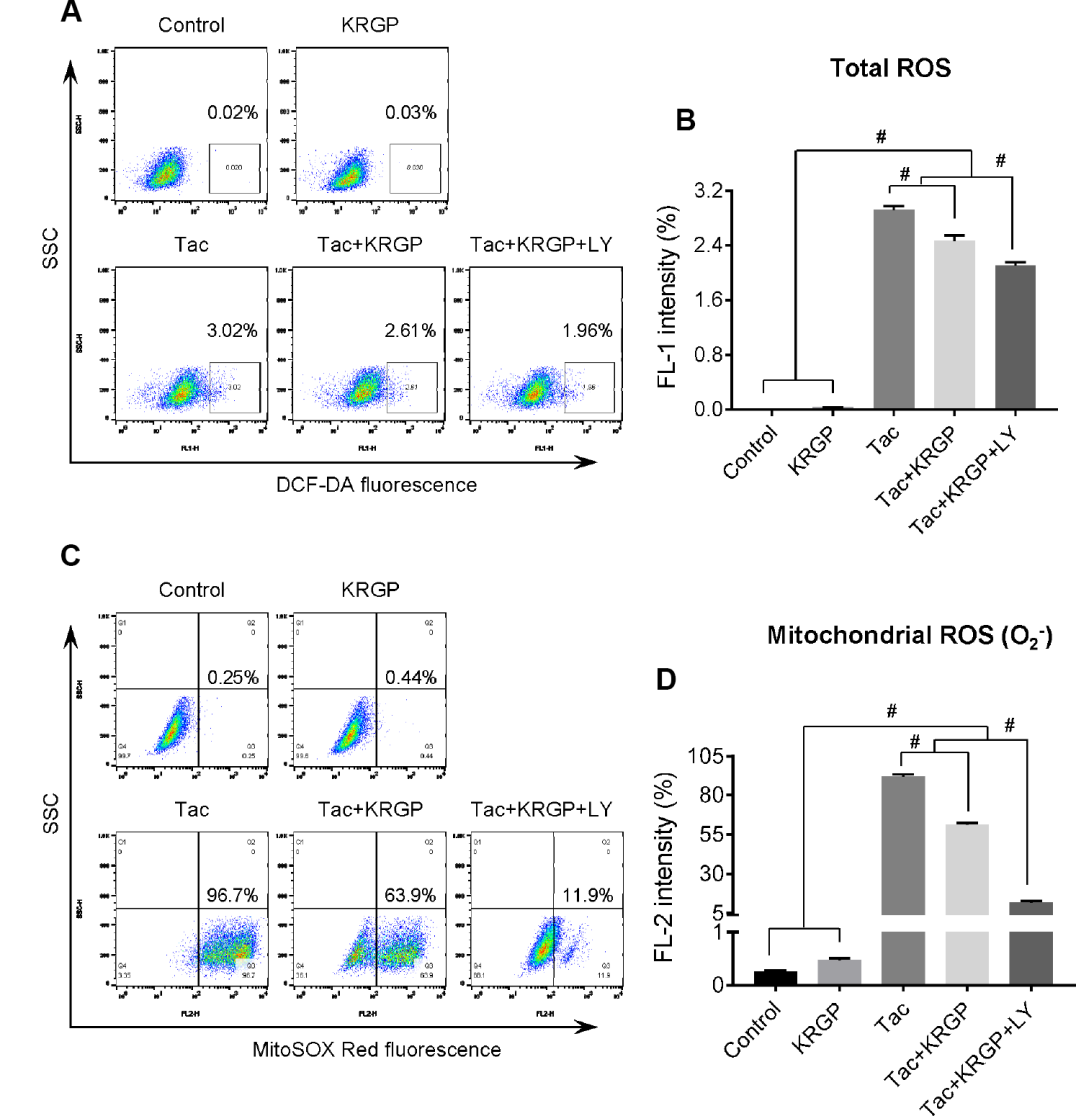
**Effect of KRGP treatment on Tac-induced ROS production in HK-2 cells.** HK-2 cells were seeded in a culture plate at 90% confluence. On the next day, the cells were treated with Tac (50 μg/mL) in the absence or presence of 10 μg/mL KRGP and 25 μM LY294002 (LY, PI3K inhibitor) for 12 h. The cells were exposed in DCF-DA or MitoSOX Red, and then analyzed by flow cytometry. (**A** and **B**) Flow cytometry plots and a quantitative graph of DCF-DA fluorescence for detecting total ROS. (**C** and **D**) Flow cytometry plots and a quantitative graph of MitoSOX Red fluorescence for detecting mitochondrial ROS (O_2_^-^). Data are presented as mean ± SE and are representative of at least three independent experiments. ^#^P < 0.05.

Next, we evaluated whether KRGP treatment decreases Tac-induced intracellular ROS accumulation, specifically mitochondrial superoxide (O_2_^-^), using flow cytometric analysis of HK-2 cells. KRGP co-treatment showed significantly reduced MitoSOX Red fluorescence (for detecting O_2_^-^) and DCF-DA fluorescence (for detecting total ROS); suppression of PI3K by LY294002 further reduced the expression (Figure 8).

### Effect of KRGP on Tac-induced mitochondrial damage and apoptosis in HK-2 cells

**Figure 9:**
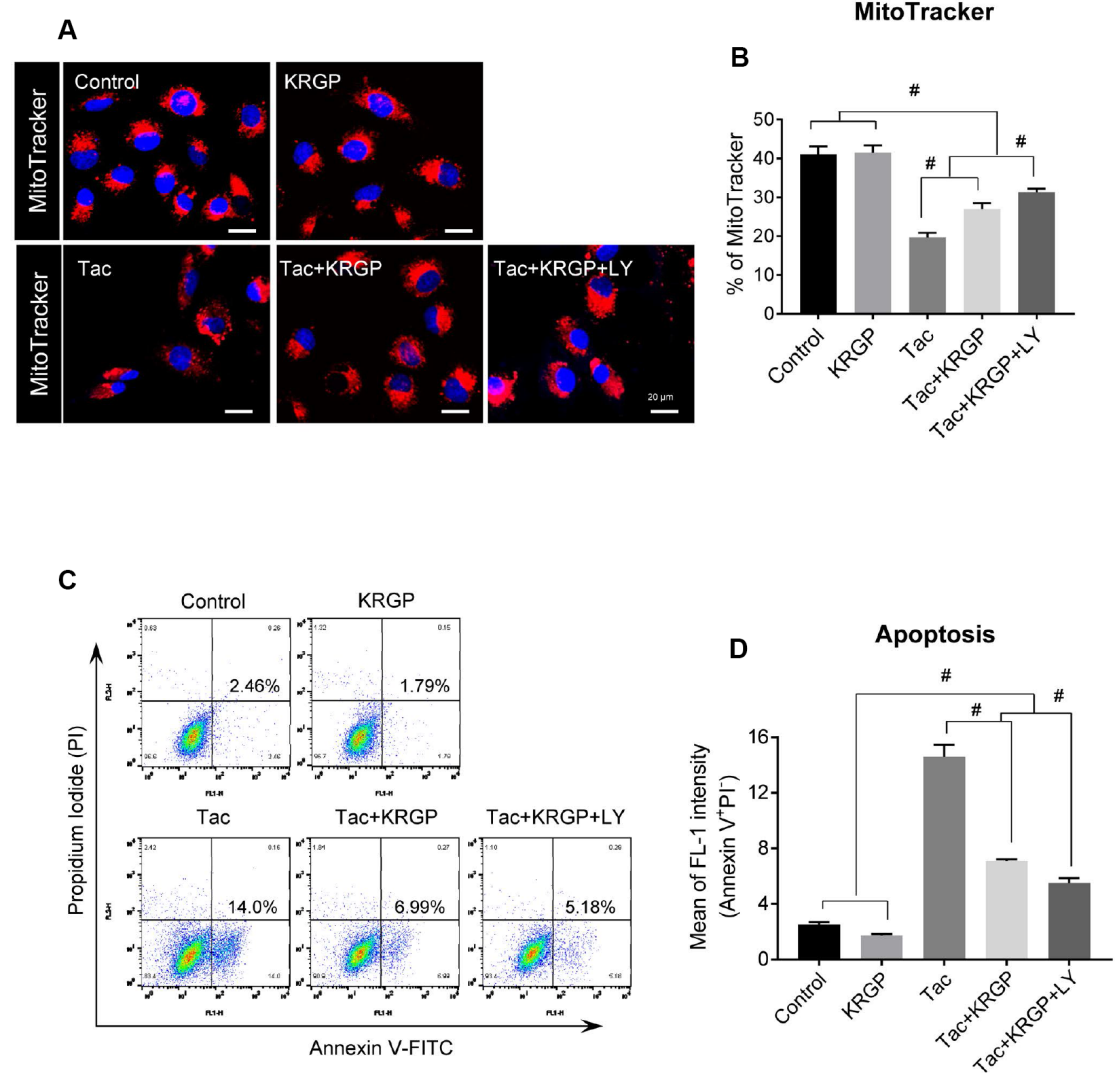
**Effect of KRGP treatment on Tac-induced mitochondrial damage and apoptosis in HK-2 cells.** HK-2 cells were seeded in a culture plate at 90% confluence. On the next day, the cells were treated with Tac (50 μg/mL) in the absence or presence of 10 μg/mL KRGP and 25 μM LY294002 (LY, PI3K inhibitor) for 12 h. The cells were exposed to MitoTracker or Annexin V-FITC and PI, and then analyzed by confocal microscopy and flow cytometry. (**A** and **B**) MitoTracker staining to detect the number of mitochondria by confocal microscopy. (**C** and **D**) Flow cytometry histograms and a graph of annexin V and PI labeling. Data are presented as mean ± SE and are representative of at least three independent experiments. ^#^P < 0.05.

We next examined whether reduced ROS levels, especially mitochondrial ROS, due to KRGP protects against Tac-induced mitochondrial damage and apoptosis. Using MitoTracker, which incorporates into healthy mitochondria, we showed the reduction of its fluorescence intensity after Tac treatment and the restoration of its fluorescence intensity by the addition of KRGP, which implied that KRGP reduced mitochondrial damage (Figure 9A and 9B).

To evaluate whether KRGP suppresses apoptosis, a mitochondria-dependent pathway, we performed flow cytometric analysis by annexin V and PI staining. The Tac group showed a higher percentage of annexin V-positive and PI-negative cells, but this high percentage was significantly reduced by KRGP, and reduced to a greater extent by LY294002 (Figure 9C and 9D).

### Effect of KRGP on Tac-induced mitochondrial dysfunction in HK-2 cells

**Figure 10:**
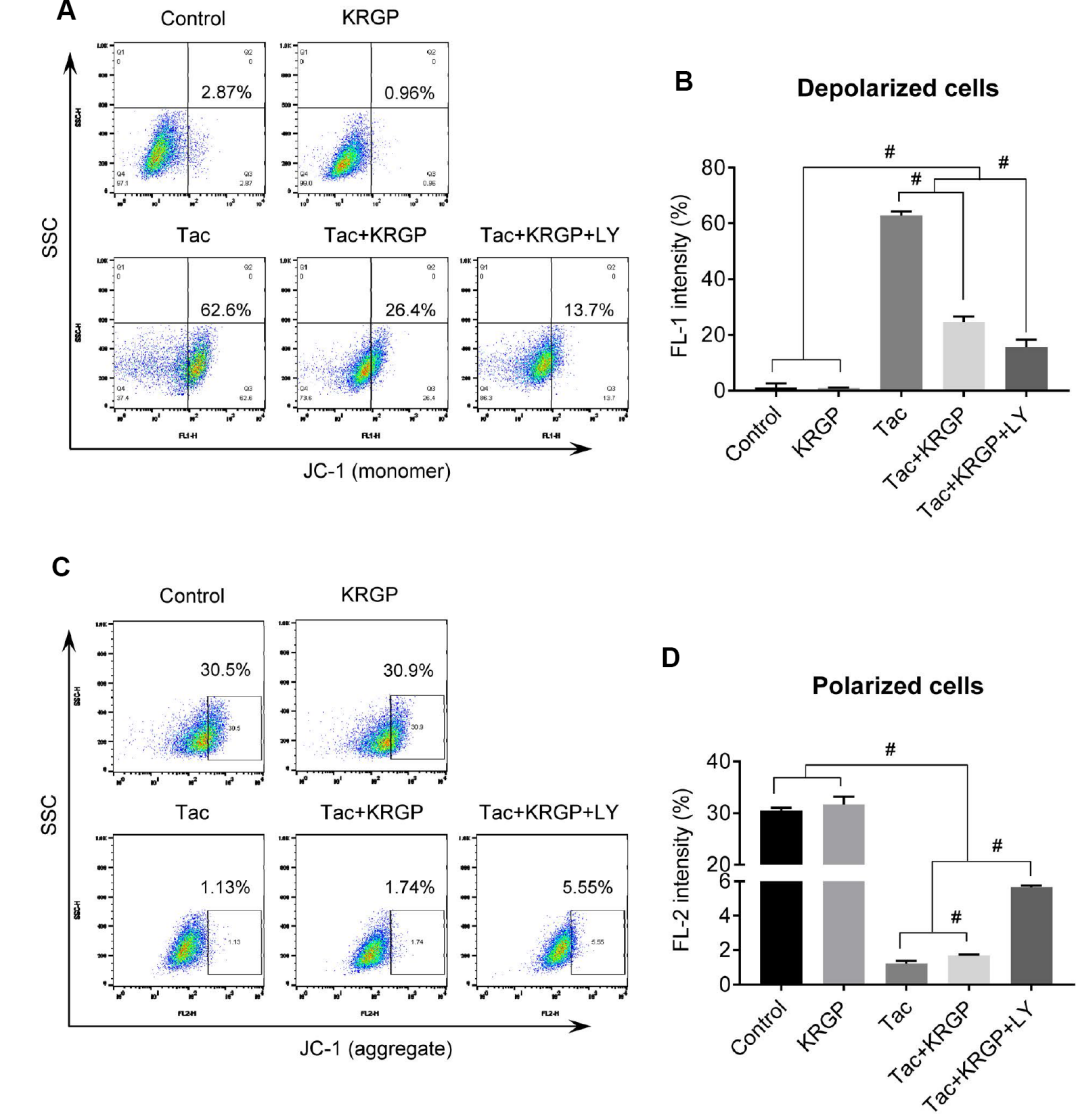
**Effect of KRGP treatment on Tac-induced impairment of MMP in HK-2 cells.** HK-2 cells were seeded in a culture plate at 90% confluence. On the next day, the cells were treated with Tac (50 μg/mL) in the absence or presence of 10 μg/mL KRGP and 25 μM LY294002 (LY, PI3K inhibitor) for 12 h. The cells were labeled with JC-1 to evaluate mitochondrial membrane potential (MMP) and then analyzed by flow cytometry. (**A** and **B**) Flow cytometry plots and a quantitative graph of JC-1 (monomer) for detecting mitochondrial polarized cells. (**C** and **D**) Flow cytometry plots and a quantitative graph of JC-1 (aggregate) for detecting mitochondrial depolarized cells. Data are presented as mean ± SE and are representative of at least three independent experiments. ^#^P < 0.05.

**Figure 11:**
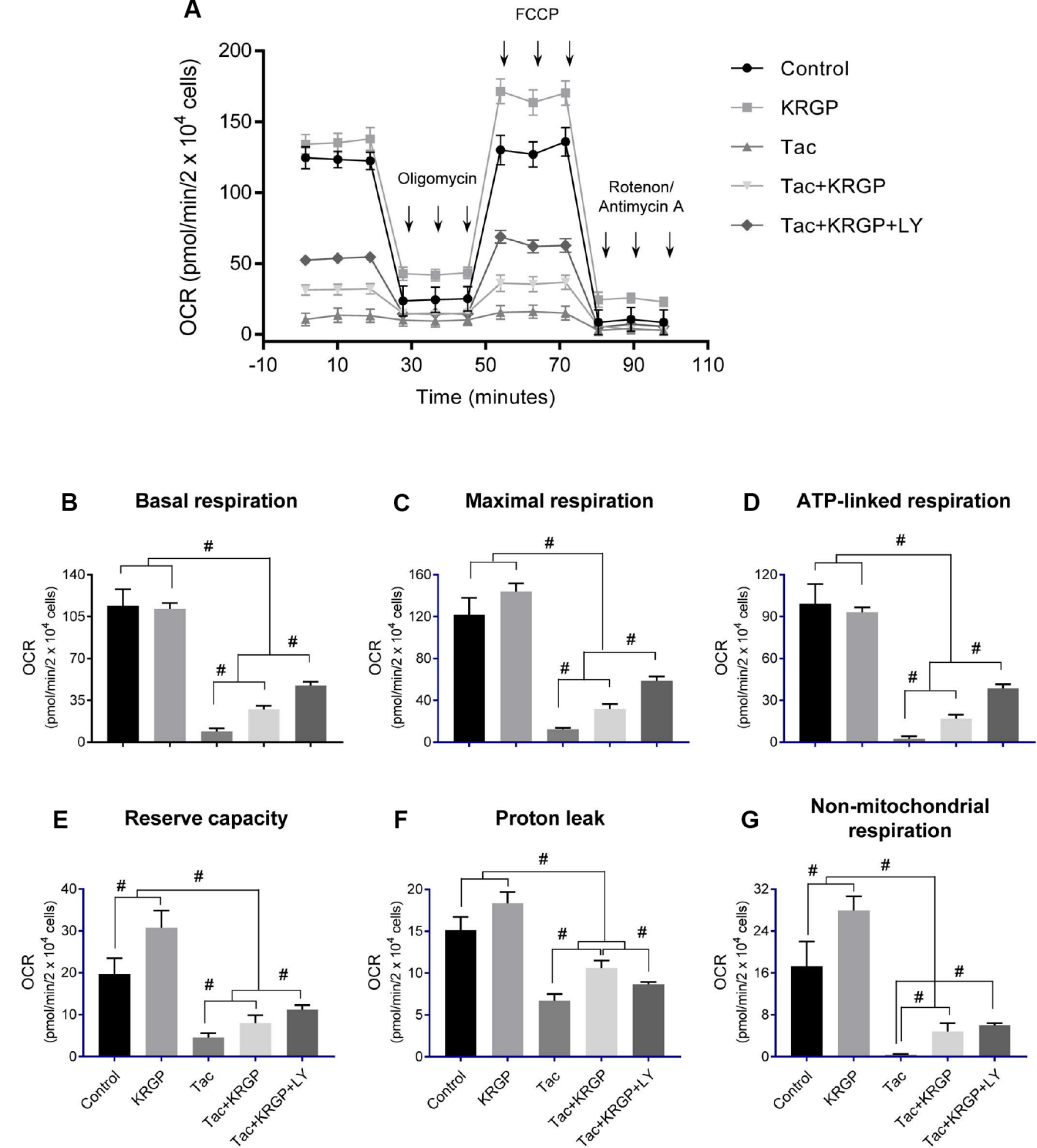
**Effect of KRGP treatment on Tac-induced impairment of mitochondrial oxygen consumption rate in HK-2 cells.** HK-2 cells were seeded in culture plates at 90% confluence. On the next day, the cells were treated with Tac (50 μg/mL) in the absence or presence of 10 μg/mL KRGP and 25 μM LY294002 (LY, PI3K inhibitor). After a 12-h treatment, the cells were incubated in a non-CO_2_ incubator for 1 h. Next, the ATP synthase inhibitor oligomycin, the uncoupler FCCP, or the respiratory chain complex I and III inhibitor rotenone/antimycin A was added to the culture medium, as indicated (**A**). The areas under the curve for basal respiration (**B**), maximal respiration (**C**), ATP-linked respiration (**D**), reserve capacity (**E**), proton leak (**F**), and non-mitochondrial respiration (**G**) were calculated from OCR. Data are presented as mean ± SE and are representative of at least three independent experiments. ^#^P < 0.05.

We examined mitochondrial membrane potential (MMP) and oxygen consumption rate after Tac and KRGP treatments in HK-2 cells. As shown in Figure 10, the significantly increased JC-1 monomer fluorescence (detecting depolarized cells) in the Tac group was reduced by treatment with KRGP. On the contrary, the reduced number of polarized cells by Tac was restored in the Tac + KRGP group. Cellular oxygen-consumption rate (OCR) was assessed, as shown in Figure 11. Tac with KRGP showed a higher OCR than that after Tac treatment alone. KRGP-co-treated cells exhibited higher basal respiration, maximal respiration, ATP-linked respiration, reserve capacity, proton leak, and non-mitochondrial respiration than the cells untreated with KRGP. LY294002 treatment with Tac + KRGP caused further increase in mitochondrial function.

## DISCUSSION

The results of the present study clearly showed the protective mechanism of ginseng against Tac-induced renal injury in a mouse model. Treatment with ginseng is associated with the promotion of Klotho and its antiaging signaling, suggestive inhibition of PI3K/AKT-induced FoxO3a phosphorylation, and subsequent enhancement of the binding of FoxO3a to the MnSOD promoter region in a setting of Tac-induced oxidative injury. These effects increased MnSOD mRNA and protein expression in the mitochondria and protected mitochondrial function. These findings suggested the profound induction of Klotho signaling by ginseng via attenuation of oxidative stress following Tac treatment; this effect is associated with improved mitochondrial function.

We have shown the significant reduction of Klotho expression in a mouse model of chronic CsA nephropathy. Indeed, Klotho heterozygous mice showed aggravated Tac-induced nephropathy [10]. Therefore, we hypothesized that the protective effect of ginseng in chronic CNI-induced renal injury is closely correlated with an increase in Klotho expression. In the current study, we revealed that co-treatment with ginseng restored the plasma and intrarenal Klotho expression, as well as improved renal function and tubulointerstitial fibrosis in a model of Tac-treated renal injury. These findings suggested that the renoprotective properties of ginseng are closely related to the action of Klotho-mediated pathway. CNI treatment is well-known to induce oxidative-stress injury by increasing the production of ROS and decreasing the activities of antioxidant enzymes [8, 9, 14, 20, 27]. Previous studies and our current data suggested that co-treatment with ginseng decreases the expression of oxidative stress marker and apoptotic cell death [28]. In addition, it was found to be effective in restoring mitochondrial number and size compared to that in the group treated with Tac alone, as observed in the current study. Therefore, we suggest that the antioxidant effect of ginseng plays an important role in reducing Tac-induced mitochondrial injury, and subsequent apoptosis. We next focused on the involvement of the Klotho signaling pathway in the promotion of the transcription of MnSOD, which is an antioxidant enzyme located in the mitochondria.

Induction of MnSOD by Klotho may contribute to various mechanisms, including protein kinase C-, PI3K/AKT, and MAP kinase-mediated pathways. Earlier, Klotho had been found to inhibit the PI3K/AKT pathway because its downstream targets comprised of those resulting from FoxO3a-mediated MnSOD expression [7, 11, 29]. Ginseng co-treatment reduced PI3K, phosphorylated AKT, and FoxO3a levels, hence implying the enhancement of the nuclear expression of FoxO3a, as shown in Figure 6. We finally observed restoration of the mRNA and protein levels of MnSOD by *in-situ* hybridization, immunohistochemical, and immunoblotting analyses following ginseng co-treatment. These data together suggested a relationship between Klotho-induced PI3K/AKT/FoxO-signaling and MnSOD following ginseng treatment, and such associations might provide renoprotection owing to Klotho-induced antioxidant effect upon Tac-stimulated oxidative injury.

Next, we studied the causality between the ginseng-induced Klotho-regulatory activity and MnSOD transcription level. *In vitro* study showed that ginseng treatment promoted nuclear FoxO3a translocation; we confirmed the PI3K/AKT pathway by LY294002 and PI3K inhibitor treatment. ChIP assays showed that addition of ginseng enhanced FoxO3a binding to the promoter region of the MnSOD gene, the latter being associated with increased MnSOD mRNA and protein levels. These results suggested that the addition of ginseng- induced Klotho signaling by inhibiting Tac-induced PI3K/AKT, thereby accelerating FoxO3a translocation to the nucleus, which in turn was stabilized through the binding of FoxO3a to the MnSOD promoter.

We explored whether ginseng-induced expression of MnSOD, a mitochondrial antioxidant enzyme, helps improve Tac-induced mitochondrial dysfunction. Both mitochondrial membrane depolarization and excessive mitochondrial ROS formation by Tac were reversed by ginseng co-treatment. In addition, mitochondrial function showed that ginseng-treated cells had significantly higher OCR levels, including basal OCR, maximal OCR, and ATP-linked OCR, than the Tac group. These favorable effects of ginseng in the mitochondria were related to the PI3K/AKT pathway, which was inhibited by LY294002. Finally, mitochondrial damage-related cell death and apoptosis, as well as the number of healthy mitochondria as confirmed by MitoTracker, were all restored by treatment with ginseng. Taken together, our results suggested that the addition of ginseng protected against Tac-induced mitochondrial function and apoptosis via Klotho-induced antioxidant signaling pathway.

In this study, we used ginseng extract instead of specific ginsenosides because extracts are one of the most convenient ways for humans to consume ginseng. However, ginseng extracts contain multiple functional constituents, making it important to identify the key components involved directly in Klotho induction. Unfortunately, little is known regarding the effect of ginsenoside components, except Rg1, on Klotho signaling in kidney diseases [30]. Therefore, we examined which of the main ginsenosides of KRGP, e.g. Rb1, Rb2, Rc, Rd, and Rg (3), contributes to Klotho expression. Compared to KRGP, most of the ginsenosides restored cell viability and Klotho protein level upon Tac-induced HK-2 cell injury (Supplementary Figures 1, 2). Based on these results, all the components examined here may be considered to have a protective effect associated with Klotho signaling, and hence may be contributing to the effect of KRGP, as observed in the current study.

**Figure 12:**
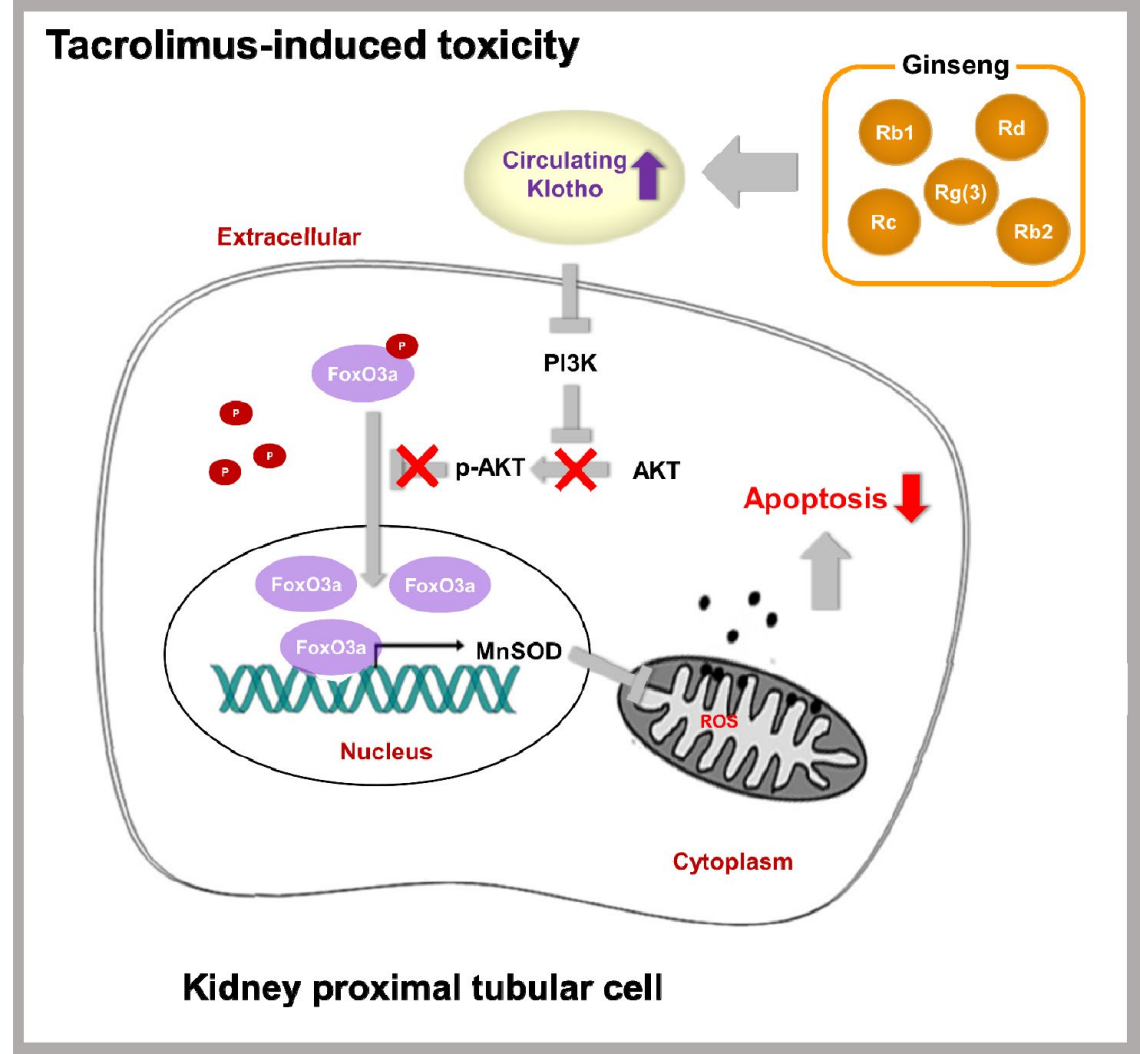
**Proposed mechanism of the protective effect of KRGP upon Tac-induced renal injury.**

Figure 12 summarizes the mechanism suggested for the protective activity of ginseng upon Tac-induced kidney damage. PI3K/AKT-mediated phosphorylation of FoxO3a was induced by reduced Klotho level following Tac treatment. FoxO3a seemed to be present in an inactive form in the cytoplasm. However, restoration of Klotho level by ginseng induced the nuclear translocation of FoxO3a by suppressing PI3K/AKT activity and increasing MnSOD level. With this mechanism, ginseng preserved Klotho expression, protecting against oxidative damage and apoptotic cell death upon Tac-induced toxicity. Therefore, our results provided evidence of the protective mechanism of ginseng via the antiaging gene Klotho in a setting of oxidative stress injury.

## MATERIALS AND METHODS

### Mouse model of Tac-induced renal injury

All experiments were carried out in agreement with the recommendations and ethical guidelines of the Animal Care and Use Committee of the Catholic University of Korea (CUMC-2017-0245-03). All experimental animals were sacrificed under xylazine (rompun) anesthesia. Eight-week-old male BALB/c mice (ORIENT BIO, Seongnam-Si, Korea) were provided 0.01% salt diet (Research Diets Inc, New Brunswick, NJ, USA) and water *ad libitum*. After 1-week acclimatization, weight-matched mice were randomly sorted into four groups (n = 8/group) and treated subcutaneously with 1.5 mg/kg/day of Tac (Prograf; Astellas Pharma, Ibaraki, Japan) or 10 mL/kg/day of vehicle (Vh, olive oil; Sigma-Aldrich, St. Louis, MO, USA), with or without Korean red ginseng powder (KRGP; 0.2 g/kg, oral gavage; Korea Ginseng Corporation, Seoul, Korea) for 4 weeks. Route of administration and doses were selected as per earlier reports [28, 31–33]. Animals were then anaesthetized, and blood samples and tissue specimens were obtained for further analysis.

### Measurement of serum creatinine (Scr)

Scr was evaluated by a quantitative enzymatic colorimetric method (0430-120; Stanbio Laboratory, Boerne, TX, USA), according to the manufacturer’s instruction.

### Detection of Klotho and 8-OHdG

Enzyme-linked immunosorbent assay (ELISA) kits were used to detect Klotho (CSB-E14362m; Cusabio Technology, Houston, TX, USA) and 8-OHdG (STA-320; Cell Biolabs, San Diego, CA, USA) in serum, according to the manufacturer’s instruction.

### Renal tubulointerstitial fibrosis

Renal histology was assessed using Trichrome-stained tissue sections, as previously described [31]. In brief, the extent of fibrosis was estimated in a minimum of 20 fields per section by counting the percentage of injured area per field using the polygon program (TDI Scope Eye, Version 3.6 for Windows; Seoul, Korea). Histopathological analysis was performed in randomly selected cortical fields by a pathologist blinded to the identity of the treatment groups.

### Antibodies

The following primary antibodies were used for immunoblot analysis or immunohistochemical staining: anti-e-cadherin (BD Biosciences, San Jose, CA, USA), α-SMA (A2547; Sigma-Aldrich), TGF-β1 (R&D systems, Minneapolis, MN, USA), anti-8-OHdG (MOG-100P; JaICA, Shizuoka, Japan), anti-Klotho (KAL-KO603; Cosmo Bio., Tokyo, Japan), anti-MnSOD (ab16953; Abcam, Cambridge, UK), anti-PI3K (610045; BD Transduction Laboratories, San Jose, CA, USA), p-AKT (Ser473) (9271S; Cell Signaling Technology, Danvers, MA, USA), t-AKT (9272S; Cell Signaling Technology), p-FoxO3a (9466S; Cell Signaling Technology), t-FoxO3a (2497S; Cell Signaling Technology), anti-β-actin (A5441; Sigma–Aldrich), and anti-COX-IV (A301-899A; Bethyl Laboratories, Montgomery, TX, USA).

### TUNEL staining

*In situ* apoptosis detection kit (S7100; Millipore Corp, Billerica, MA, USA) was used to detect apoptotic cells and count TUNEL-positive cells in approximately 20 randomly selected non-overlapping areas per animal per group.

### Electron microscopy

After fixation in 2.5% glutaraldehyde and 0.1 M phosphate buffer, renal cortical tissues were post-fixed with 1% O_S_O_4_ and embedded in Epon 812. Ultrathin sections were cut, stained with uranyl acetate/lead citrate, and photographed with a JEM-1200EX transmission electron microscope (JEOL Ltd., Tokyo, Japan). Sections were scanned randomly at 20 different spots per sample at 5000× magnification. The numbers and sizes of mitochondria were measured in 20 random proximal tubular cells, using an imaging software (TDI Scope Eye).

### Immunohistochemical analysis

De-waxed sections were incubated in retrieval solution (pH 6.0), methanolic H_2_O_2_, and 0.5% Triton X-100, and subsequently washed in phosphate-buffered saline. Non-specific binding sites were blocked with 10% normal donkey serum (Jackson ImmunoResearch, West Grove, PA, USA). Sections were incubated with primary antibodies at 4 °C overnight and then with peroxidase-conjugated secondary antibody (Jackson ImmunoResearch) at room temperature (RT) for 2 h. Peroxidase activity was detected using 3,3′-diaminobenzidine (DAB; Vector Laboratories, Burlingame, CA, USA) as a chromogen. Stained tissues were viewed using an Olympus photomicroscope equipped with differential-interference contrast optics (BX 51; Olympus, Tokyo, Japan). Quantitative analysis was performed by calculating the percentage of positive area showing the same intensity using histogram equalization (TDI Scope Eye).

### *In-situ* hybridization

MnSOD mRNA transcripts in tissues were visualized according to the instructions of the manufacturer of the RNAScope® 2.5 HD Detection Kit (322371; Advanced Cell Diagnostics, Newark, CA, USA), using a validated probe for mouse MnSOD (439361; Advanced Cell Diagnostics). Briefly, 5-μm slide-mounted sections were heated for 60 min at 60 °C in a HybEZ^TM^ hybridization oven (Advanced Cell Diagnostics). Tissues were de-waxed in xylene, followed by dehydration in an ethanol series and air drying for 5 min. Tissue sections were incubated with pretreatment 1 solution (endogenous peroxidase block) for 10 min at RT. Slides were rinsed by immersion in double-distilled water (DW), followed by immersion in pretreatment 2 solution (antigen-retrieval citrate buffer) for 15 min at 100–104 °C. Slides were washed again in DW before pretreatment 3 (protease) solution was applied for 30 min at 40 °C. Slides were re-washed in DW, and target or control probes were incubated at 40 °C for 2 h followed by rinsing in wash buffer for 2 min at RT. Signal-amplification reagents 1 to 6 were applied sequentially for 30 min and 15 min, 30 min, 15 min, 30 min, and 15 min, respectively. Slides were rinsed in wash buffer for 2 min between amplification reagents. Incubations with amplifier reagents 1 to 4 were conducted at 40 °C, whereas those with amplifier reagents 5 and 6 were conducted at RT. Positive signals were visualized using DAB (Vector Laboratories). After drying for 15 min at 60 °C, coverslips were placed on the slides using mounting medium (SP15-100; Fisher Scientific Medford, MA, USA). The positive control probe consisted of a proprietary probe for *Bos taurus* cyclophilin B, whereas the negative control probe targeted dapB of *Bacillus subtilis*. Quantitative analysis was performed by calculating the percentage of positive area showing the same intensity using histogram equalization (TDI Scope Eye).

### Immunoblot analysis

Renal cortex or whole cells were lysed in 10 mM Tris (pH 7.5) containing 1% sodium dodecyl sulfate (SDS) and 1 mM NaVO_4_. Equal amounts of protein were subjected to immunoblot analysis using primary antibodies. Signals were detected using an enhanced chemiluminescence system (ATTO Corp., Tokyo, Japan). Quantification of relative intensities was performed with the control group set at 100%; densities were normalized to that of β-actin bands from the same gel (Quantity One version 4.4.0; Bio-Rad, Hercules, CA, USA).

### Cell culture

HK-2 cells, from an immortalized human proximal tubular epithelial cell line, were grown in Dulbecco’s modified Eagle medium (DMEM) containing 10% fetal bovine serum supplemented with 100 U/mL penicillin and 100 μg/mL streptomycin, and incubated at 37 °C in a humidified atmosphere containing 5% CO_2_. The cells were seeded in culture plates and treated with Tac (50 μg/mL) and KRGP (10 μg/mL, Korea Ginseng Corporation), with or without LY 294002 (25 μM, Sigma-Aldrich) for 12 h.

### Cell-viability assay

Cells were seeded in 96-well plates at a density of 2 x 10^4^ cells/well for 24 h and then subjected to various treatments for the specified durations. Before the end of the treatments, Cell Counting Kit (CCK)-8 solution (Dojindo Molecular Technologies, Kumamoto, Japan) was added to each well for 2 h. Absorbance was measured at 450 nm using a VersaMax ELISA Reader (Molecular Devices, Sunnyvale, CA, USA).

### Immunofluorescence for FoxO3a

Cells were fixed with cold 4% paraformaldehyde for 15 min and washed thrice in phosphate-buffered saline. Thereafter, they were blocked in 10% normal donkey serum for 1 h at RT, and incubated with anti-t- FoxO3a (Cell Signaling Technology) at 4 °C overnight. Subsequently, the cells were incubated with Cyanine 3 (Cy^3^)-conjugated secondary antibodies (Jackson ImmunoResearch) for 2 h at RT. Nuclei were viewed with 4′,6-diamidino-2-phenylindole (DAPI; Vector Laboratories) after 5-min incubation at RT. Cells were imaged using a Zeiss LSM700 confocal microscope (Carl Zeiss Microscopy GmbH, Jena, Germany). The positive nuclear FoxO3a counting in each group was calculated based on 100% control

### Mitochondrial fractionation

Cells were harvested using a scraper and centrifuged at 500 x *g*. The cell pellet was washed by resuspension in phosphate-buffered saline. Mitochondria was separated by using a Mitochondria Isolation Kit (Pierce Biotechnology, Rockford, IL, USA). Mitochondrial fractions were confirmed using COX-IV antibody (A301-899A; Bethyl Laboratories).

### ChIP experiments

ChIP was performed using a commercial kit (17-295; Millipore Corp). Briefly, cells were incubated with or without KRGP for 12 h following Tac treatment, after which they were cross-linked with 1% formaldehyde. After washing, the cells were sonicated and immunoprecipitated with an anti-FoxO3a antibody (ab12162; Abcam) overnight at 4 °C. After elution and reverse crosslinking of the antibody/DNA complexes, cellular DNA was purified using a DNA purification kit (28104; Qiagen, Hilden, Germany) and analyzed by q-PCR using primer pairs covering the MnSOD promoter region containing a FoxO3a-binding element at position -1,249 (5′-GAG TAT CTA TAA CCT GGT CCC AGC C-3′ and 5′-GCT GAA CCG TTT CCG TTG CTT CTT GC-3′). Data are shown as the amount of DNA relative to the input.

### qRT-PCR

Total RNA from cultured cells was isolated using RNA-Bee (CD-105B; Tel-Test, Friendswood, TX, USA). First-strand cDNA was synthesized and subjected to quantitative real-time PCR (q-PCR) using SYBR Green Master Mix in a LightCycler 480 system (04707516001; Roche, Rotkreuz, Switzerland). Gene expression was normalized to cyclophilin A expression using the change-in-threshold method and primers with the sequences 5′-GGTCCCAAAGACAGCAGAAA-3′ and 5′-GTCACCA CCCTGACACATAAA-3′.

### Determination of mitochondrial damage

Mitochondrial integrity was assessed using MitoTracker Deep Red FM (M22426; Invitrogen, Carlsbad, CA, USA). After treatment with the drug or vehicle for 20 min at 37 °C, as per the manufacturer’s instructions, cells were analyzed using a FACS Calibur flow cytometer (BD Biosciences) or under an LSM700 confocal microscope (Carl Zeiss Microscopy GmbH).

### Apoptosis

After treatment with the drug or vehicle, trypsinized cells were incubated with 5 μL of fluorescein isothiocyanate-conjugated annexin V (556419; BD Biosciences) and propidium iodide (PI, 556463; BD Biosciences) in 1× binding buffer (556454; BD Biosciences) for 15 min at RT, according to the manufacturer’s protocol. Stained cells were analyzed by flow cytometry on a FACS Calibur instrument (BD Biosciences). Values are expressed as the percentage of fluorescent cells relative to the total cell count.

### ROS generation

After treatment with the drug or vehicle, intracellular total ROS and mitochondrial ROS (superoxide anion) were detected using H_2_DCF-DA (Molecular Probes, Carlsbad, CA, USA) and MitoSox Red (Invitrogen) for 30 min at 37 °C, respectively, according to the manufacturer’s instructions, and analyzed using either a FACS Calibur flow cytometer (BD Biosciences) or an LSM700 confocal microscope (Carl Zeiss Microscopy GmbH). Both forward and side scatter data were collected (10,000 events per sample).

### Mitochondrial membrane potential (MMP)

The MMP of HK-2 cell was measured using the fluorescent, lipophilic cationic probe, JC-1 (Cayman, Ann Arbor, MI, USA). When live cells are incubated with JC-1, the dye penetrates the cell membrane as monomers and this uptake into mitochondria is driven by the MMP. Functional mitochondria are polarized, because of which JC-1 is rapidly taken up. This uptake raises the JC-1 concentration leading to the formation of aggregates (J-aggregates) within mitochondria. When excited by a 488-nm laser, J-aggregates provoke a red shifted emission at 590 nm. In depolarized mitochondria, JC-1 does not accumulate; it rather remains in the cytoplasm as monomers, which emit at 525 nm. Thus, in healthy cells, JC-1 fluorescence (monomer) was seen in FL-1 channels, and JC-1 fluorescence (aggregate) in FL-2 channels indicated depolarized mitochondria. After treatment with the drug or vehicle, the cells were incubated with JC-1 staining solution for 20 min at 37 °C, according to the manufacturer’s instructions, and then analyzed using either a FACS Calibur flow cytometer (BD Biosciences) or an LSM700 confocal microscope (Carl Zeiss Microscopy GmbH).

### OCR experiments

Cellular OCR rate was assessed in real-time using an XF24 extracellular flux analyzer (Seahorse Biosciences, Billerica, MA, USA). After treatment with the drug or vehicle, the cell medium was changed to running medium (DMEM supplemented with 5.5 mM glucose, 1 mM sodium pyruvate, 4 mM L-glutamine, pH 7.4), and incubated at 37 °C in a non-CO_2_ incubator for 1 h. ATP synthase inhibitor oligomycin (1 μM), carbonyl cyanide-4 (trifluoromethoxy) phenylhydrazone (FCCP; 0.5 μM), and the complex I and III inhibitor rotenone/antimycin A (0.5 μM) were used as the mitochondrial inhibitors. OCR values were subsequently normalized to the protein content of each sample. Mitochondrial function-related parameters were determined using these mitochondrial inhibitor compounds as modulators to determine several bioenergetics and mitochondrial function parameters, including basal respiration, ATP production, maximal respiration, and spare respiratory capacity, as described by Dott et al [34]. Three to four wells were used for each experimental group.

### Statistical analyses

Data are expressed as the mean ± standard error (SE) from at least three independent experiments. Multiple comparisons between groups were performed by one-way analysis of variance with Bonferroni *post-hoc* test, using the Prism software (Version 7.03 for Windows, GraphPad Software, La Jolla, CA, USA). Results with P values < 0.05 were considered statistically significant.

## Supplementary Material

Supplementary Figures
