# Supplementary material for: Ginseng increases Klotho expression by FoxO3-mediated manganese superoxide dismutase in a mouse model of tacrolimus-induced renal injury
Source: Aging (Albany NY). 2019 Aug 10;11(15):5548–69. doi: 10.18632/aging.102137 (PMC6710054; doi:10.18632/aging.102137)
Supplement: Supplementary Figures [file aging-11-102137-s001.pdf]

SUPPLEMENTARY MATERIAL

Supplementary Figures

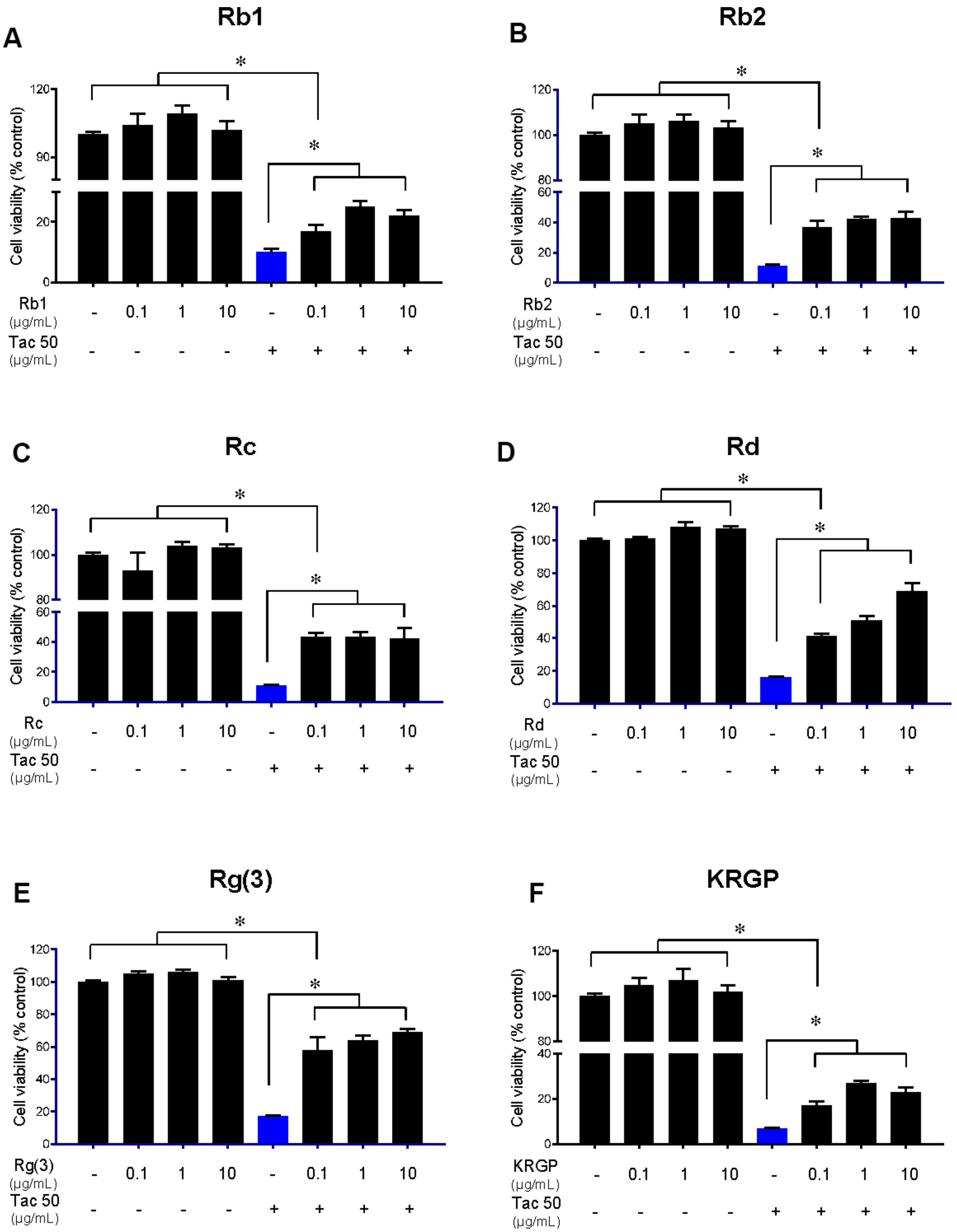

Supplementary Figure 1. Comparing the effect of Rb1, Rb2, Rc, Rd, Rg(3), and KRGP on the cell viability in Tac-treated HK-2 cells. Cell viability of HK-2 cells detected using CCK-8 assay at 0.1, 1, and 10 µg/mL of (**A**) Rb1, (**B**) Rb2, (**C**) Rc, (**D**) Rd, (**E**) Rg(3), and (**F**) KRGP during Tac-induced injury for 12 h. Data are presented as mean ± SE and are representative of at least three independent experiments. $^{*}P < 0.05$ vs. indicated groups.

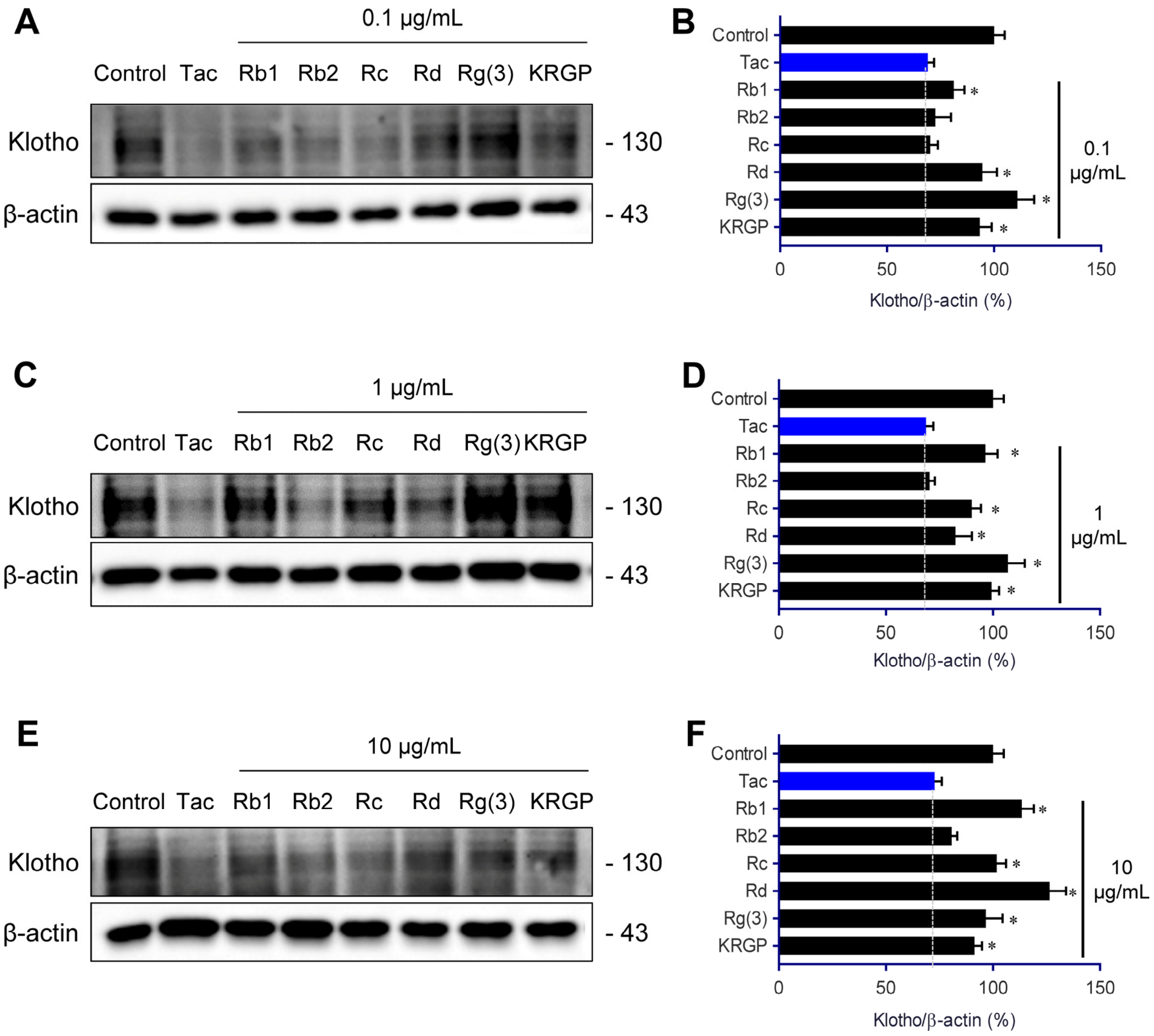

Supplementary Figure 2. Comparing the effect of Rb1, Rb2, Rc, Rd, Rg(3), and KRGP treatment on the Klotho expression in Tac-treated HK-2 cells. Representative immunoblot image and its quantification of Klotho during Tac and 0.1 (**A** and **B**), 1 (**C** and **D**), 10 ug/mL (**E** and **F**) of Rb1, Rb2, Rc, Rd, Rg(3), and KRGP treatment for 12 h. The relative optical densities of bands in each lane were normalized to each band of β-actin or total form protein from the same gel. Data are presented as mean ± SE and are representative of at least three independent experiments. *$P < 0.05$ vs. Control group.

Supplementary Methods

Cell culture

HK-2 cells from an immortalized human proximal tubular epithelial cell line were grown in Dulbecco's modified Eagle's medium (DMEM) containing 10% fetal bovine serum supplemented with 100 U/mL penicillin and 100 µg/mL streptomycin, and incubated at 37°C in a humidified atmosphere containing 5% CO_2. The cells were seeded in culture plates and treated with Tac (50 µg/mL) and Rb1, Rb2, Rc, Rd, Rg(3), and KRGP (0.1, 1, and 10 µg/mL, Korea Ginseng Corporation) for 12 h.

Cell-viability assay

Cells were seeded in 96-well plates at a density of 2 x 10^4 cells/well for 24 h and then subjected to various treatments for 12 h. Before the end of the treatments, Cell Counting Kit (CCK)-8 solution (Dojindo Molecular Technologies, Kumamoto, Japan) was added to each well for 2 h. Absorbance was measured at 450 nm using a VersaMax ELISA Reader (Molecular Devices, Sunnyvale, CA, USA).

Immunoblot analysis

After the end of the treatment of drugs, whole cells were lysed in 10 mM Tris (pH 7.5) containing 1% sodium dodecyl sulphate (SDS) and 1mM $NaVO_4$. Equal amounts of protein were subjected to immunoblotting analysis with anti-Klotho antibody (KAL-KO603; Cosmo Bio., Tokyo, Japan). Signals were detected using an enhanced chemiluminescence system (ATTO Corp., Tokyo, Japan). Quantification of relative densities was performed with the control group set at 100%; densities were normalized to that of β-actin bands from the same gel (Quantity One version 4.4.0; Bio-Rad, Hercules, CA, USA).

Statistical analyses

The data are expressed as the mean ± standard error from at least three independent experiments. Multiple comparisons between groups were performed by one-way analysis of variance with Bonferroni post-hoc test, using Prism software (Version 7.03 for Windows, GraphPad Software, La Jolla, CA, USA). Results with P values < 0.05 were considered significant.
